# Clinical characteristics and outcomes of children with dilated cardiomyopathy in a tertiary cardiac center: a five-year review

**DOI:** 10.3389/fcvm.2026.1897834

**Published:** 2026-08-18

**Authors:** Zawadi Edward Kalezi, Stella Mihayo Mongella, Lulu Said Fundikira, Deogratias Arnold Nkya, Vivienne Aiyana Mlawi, Sulende Daudi Kubhoja, Alphonce Nsabi Simbila

**Affiliations:** 1 Department of Pediatric Cardiology, Jakaya Kikwete Cardiac Institute, Dar es Salaam, Tanzania; 2 Muhimbili University of Health and Allied Sciences, Dar es Salaam, Tanzania; 3 Emergency Medicine Department, Muhimbili National Hospital, Dar es Salaam, Tanzania

**Keywords:** arrhythmias, cardiomyopathy, children, myocarditis, Tanzania

## Abstract

**Background:**

Dilated cardiomyopathy (DCM) represents the commonest form of cardiomyopathy in children, significantly contributing to morbidity and mortality among those affected. Nevertheless, in developing countries, there is still limited published data on DCM, especially in the pediatric population. Therefore, this study aimed to document the clinical characteristics and treatment outcomes of children with DCM.

**Methods:**

This was a retrospective study of children with DCM admitted to the Jakaya Kikwete Cardiac Institute (JKCI) from January 2021 through December 2025. Socio-demographics, clinical characteristics, and survival data were extracted from the medical records. Frequencies and proportions were calculated for categorical variables. The mean with standard deviation (SD) and median with an interquartile range (IQR) were calculated for continuous data.

**Results:**

In a period of 5 years, out of 3,330 admissions, data from 35 children (1.1% 95% CI 0.73–1.46) diagnosed with DCM were analyzed. Two-thirds (68.6%) were aged below 5 years, with a slight predominance of male children. The most frequent known causes of DCM were previous clinical diagnosis of myocarditis, 42.9% (15/35), followed by arrhythmias, 14.3% (5/35), with a median age at diagnosis of 20 months (IQR, 8–60). The median left ventricular ejection fraction (LVEF) at the time of diagnosis was 30% (IQR, 25–39). During the hospital stay, 14.3% (5/35) of children developed complications; two children had a stroke while three developed acute kidney injury (AKI). The median follow-up time during clinic visits was 18 months, with a maximum time of 5 years and 9 months. At the follow-up clinic, 25% of children (7/28) recovered to normal heart function, while 35.7% (10/28) of children had unchanged LVEF. A total of nine children died; three (8.6% 95% CI 1.8–23.06) were in-hospital, and 6 (21.4% 95% CI 8.3–40.95) post-discharge. Five children (14.3%) were re-hospitalized due to exacerbation of heart failure.

**Conclusion:**

Children aged below 5 years were largely affected in our cohort, with clinically diagnosed myocarditis as the most common underlying cause. A quarter of the children showed complete recovery of heart function during the follow-up visits. We observed mortality and in-hospital complications such as stroke and AKI. Further studies involving genetic screening for the unknown etiologies of DCM are highly recommended.

## Background

Dilated cardiomyopathy (DCM) is a heart muscle disorder characterized by a left ventricular (LV) dilatation and systolic dysfunction resulting in heart failure (1). It represents the commonest form of cardiomyopathy in children, significantly contributing to morbidity and mortality among affected children (1–3). In North America, the estimated incidence of DCM is 0.57 cases per 100,000 children per year, with a higher incidence observed among infants and young children (1, 4, 5). In Africa, for instance, in Cameroon, an eighth-year retrospective study documented that 6.5% of children had DCM out of 1,666 children diagnosed with heart diseases (6). In Tanzania, at a national cardiac referral center, DCM accounts for about 4.6% of all heart disease diagnoses among children (7).

The etiologies of paediatric DCM are varied and include genetic mutations, infections (myocarditis), inborn errors of metabolism, neuromuscular diseases, and arrhythmias. However, many cases remain idiopathic (1, 4, 8–12). Regardless of the cause, DCM advances to chronic heart failure and is linked with a poor prognosis, with a significant number of affected children either dying or eventually requiring a heart transplant. In addition, a cross-sectional study from the Netherlands found an association between emotional or behavioral problems and DCM among the affected children (13).

Echocardiography (ECHO) is one of the cornerstone tools for making a diagnosis and monitoring the response to treatment of DCM. It enables evaluation of ventricular dimensions and systolic function, including fractional shortening (FS), left ventricular ejection fraction (LVEF), and end diastolic diameter (LVEDD) (1, 4, 8). The treatment of DCM focuses mainly on heart failure management due to limited disease-specific therapy, while emphasizing the prevention of complications such as arrhythmias and thromboembolism (1, 4, 9, 10). Nonetheless, in 2023, the American Heart Association proposed treatment modalities for cardiomyopathy in children, based on the clinical milieu of the patients (11). Complete recovery from DCM has been reported to be uncommon; however, a cardiomyopathy registry from the USA and Canada reports that 22% of children with DCM recovered to normal heart function within 2 years of diagnosis (11, 12).

Outcomes of pediatric DCM greatly vary between high-income countries (HICs) and developing settings. In resource-limited settings like Tanzania and many other parts of Africa, limited access to diagnostic services, medications, cardiac and intensive care contributes to delayed optimal treatment and consequently poorer outcomes. Furthermore, limited data are available on the clinical profile and hospital outcomes of pediatric DCM across many African settings (13). This study, therefore, aimed to describe the clinical characteristics and treatment outcomes of children admitted with dilated cardiomyopathy at the Jakaya Kikwete Cardiac Institute from January 2021 to December 2025.

## Methods

### Study design and setting

This was a hospital-based retrospective descriptive study conducted among children with DCM admitted to the Jakaya Kikwete Cardiac Institute (JKCI) from January 2021 through December 2025. JKCI is a 157-bed-capacity national referral and teaching center located in Dar es Salaam, Tanzania. It provides specialized cardiac services including percutaneous interventions and open cardiac surgery for both children and adults.

### Study population and participants

All children diagnosed with DCM by transthoracic echocardiography (TTE), aged 1 month to 18 years, admitted to the pediatric unit during the study period were enrolled. However, participants with incomplete data were excluded during the chart review. The patients’ electronic data were accessed for research purposes from 1st November 2025 through December 2025.

### Study variables and measurement

Medical records of all children with DCM admitted to JKCI within 5 years (January 2021 to December 2025) were reviewed. Clinical and sociodemographic parameters were age, gender, date of admission and discharge, prior history of myocarditis (based on documented clinical symptoms and echocardiography) or cardiac surgery, vitals during admission, laboratory findings, ECHO findings, documented cardiac and non-cardiac diagnoses, age at DCM diagnosis, resolution of heart failure symptoms (improvement of heart failure symptoms class), observed complications and survival (death/ alive) at discharge and during clinic visits.

The observed complications (such as acute kidney injury, stroke) were determined during the hospital stay until discharge, while mortality was determined at discharge and during scheduled follow-up visits.

Echocardiography: two-dimensional, color, pulse wave and continuous wave imaging was performed by a pediatric cardiologist using two echocardiography machines, Vivid E95 GE and Vivid^TM^ iq.

During hospital admission, every child was evaluated by a paediatric cardiologist, and echocardiography was performed. Children were considered to have DCM if echocardiography demonstrated a dilated left ventricle (LV) accompanied by systolic dysfunction and clinical signs and symptoms of heart failure. LV systolic dysfunction was defined as LVEF <55% on M-Mode (14, 15). For the follow-up echocardiography, LVEF was documented as normal (>55%), unchanged (similar values during admission and follow-up), and improved (<55% but increased compared to the admission value).

### Data management and analysis

Data entry and cleaning were done using the Statistical Package for Social Sciences (SPSS) version 25. The mean with standard deviation (SD) and median with an interquartile range (IQR) were calculated for continuous, normally distributed, and skewed data, respectively. Frequencies and proportions were calculated for categorical variables. For the missing data, the case deletion approach was used.

### Ethical consideration

Ethical approval to conduct the study was obtained from the Ethics Review Committee of the Jakaya Kikwete Cardiac Institute with ethical clearance number AB.123/307/01Q/65. All the data were anonymized to ensure patient confidentiality. Since this was a retrospective study, the ethics committee waived the requirement for informed written consent from each study participant.

## Results

**Figure 1:**
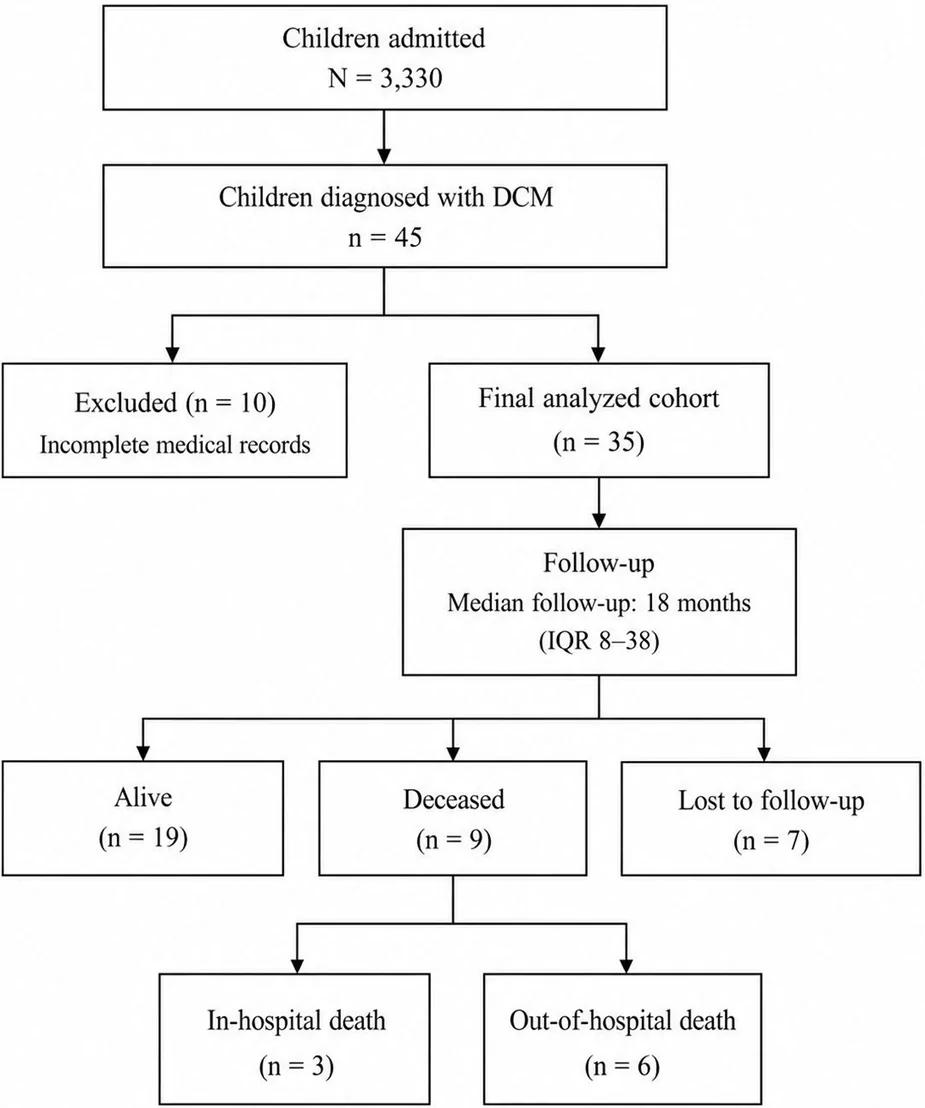
Flow diagram showing the study participants and their outcomes among children with dilated cardiomyopathy admitted to the Jakaya Kikwete cardiac institute (JKCI), Tanzania, January 2021–December 2025. Of the 3,330 children admitted during the study period, 45 were diagnosed with dilated cardiomyopathy (DCM). Ten children were excluded because of incomplete medical records, resulting in a final cohort of 35 children. The median follow-up time was 18 months (IQR, 8–38 months). At the end of the study, 19 children were alive, nine had died (three in-hospital deaths and six out-of-hospital deaths), and seven were lost to follow-up.

Within a period of 5 years, a total of 3,330 children were admitted to the JKCI; of these, 45 children (1.4%, 95% CI 0.99–1.8) had DCM. Ten children with missing information were excluded; thus, 35 (1.1%) study participants were included in the final analysis, as shown in Figure 1.

Out of 35 children with DCM, two-thirds, 24 (68.6%), were aged below 5 years, with a slight predominance of male children, 18 (51.4%). The most frequent known causes of DCM were previous clinical diagnosis of myocarditis, 15 (42.9%), and arrhythmias, 5 (14.3%), of which four children had supraventricular tachycardia. The median age at diagnosis was 20 months (IQR, 8–60); however, more than one-third of children, 14 (40%), had an unknown underlying cause of DCM. All enrolled children underwent echocardiography, and the median ejection fraction at the time of diagnosis was 30% (IQR, 25–39). Most of the admitted children received diuretic therapy, angiotensin-converting enzyme inhibitors (ACEI), and digoxin; however, two-thirds were also kept on inotropic support and beta blockers. Of those with arrhythmias, two were kept on oral propranolol and the other three were on amiodarone. Notably, 32 (91.4%) of the children had heart failure resolution at the time of discharge, and the median hospital stay was 7 days (IQR, 4–13), as shown in Table 1.

**Table 1:** Clinical characteristics and treatment of children with DCM admitted at the JKCI in Tanzania, January 2021–December 2025 (*N* = 35).

Variable	Category	Frequency (%)
Age (yr)	<1	4 (11.4)
	1–<5	24 (68.6)
	≥5	7 (20)
Gender	Male	18 (51.4)
Underlying cause of cardiomyopathy	Previous clinical diagnosis of myocarditis	15 (42.9)
	Arrhythmias	5 (14.3)
	-SVT	4 (80)
	-Frequent PVCs	1 (20)
	ALCAPA	1 (2.9)
	Idiopathic (unknown)	14 (40)
Age at the time of DCM diagnosis (months)	Median (IQR)	20 (8–60)
Left ventricular ejection fraction at time of diagnosis (percentages)	Median (IQR)	30 (25–39)
Treatment	Diuretic therapy	34 (97.1)
	^a^ACE inhibitors	33 (94.3)
	Inotropic support	11 (31.4)
	Beta blocker	14 (40)
	Digoxin	31 (88.6)
	Propranolol	2 (5.7)
	Amiodarone	3 (8.6)
Resolution of heart failure	Yes	32 (91.4)
Length of hospital stay (days)	Median (IQR)	7 (4–13)

a ACE, angiotensin converting enzymes; SVT, supraventricular tachycardia; PVCs, premature ventricular contractions; ALCAPA, anomalous left coronary artery from the pulmonary artery; IQR; interquartile range; DCM, dilated cardiomyopathy.

As per Table 2, the median follow-up time at clinic visits was 18 months (IQR, 8–38), with a maximum of 5 years and 9 months. During the follow-up, 7 (25%) children recovered to normal heart function, while 10 (35.7%) children had unchanged LVEF. Of note, 14.3% (5/35) of children developed complications; two children had a stroke, while three developed acute kidney injury. A total of nine children died, three (8.6%) during the hospital stay, while six (21.4%) died after discharge. In addition, five (14.3%) children had readmissions due to exacerbation of heart failure.

**Table 2:** Outcomes of admitted children with DCM at JKCI in Tanzania, January 2021–December 2025 (*N* = 35).

Variable	Category	Frequency (%)
Clinical complications	Yes	5 (14.3)
	Acute kidney injury	3 (8.6)
	Stroke	2 (5.7)
Ejection fraction during follow-up clinics	Normal	7 (25)^a^
	Improved	11 (39.3)^a^
	Unchanged	10 (35.7)^a^
	Lost follow-up	7 (20)
Readmissions	Yes	5 (14.3)
Mortality	In-hospital	
	Yes	3 (8.6)
	No	32 (91.4)
	Out-of-hospital	
	Yes	6 (21.4)^a^
	No	22 (78.6)^a^
	Lost to follow-up	7 (20)
Follow-up time (months)	Median (IQR)	18 (8–38)

IQR, interquartile range; DCM, dilated cardiomyopathy.

a Percentages were calculated among the 28 children with available follow-up data.

## Discussion

This study summarizes the findings of children with an echocardiographic diagnosis of DCM, admitted to JKCI within a period of 5 years. Children aged below 5 years were largely affected, and the most common cause of DCM was myocarditis. Half of the children had LVEF of 30% and below, with a quarter of affected children recovering to normal heart function during the follow-up clinics. Nonetheless, nine children died, and five were re-hospitalized due to exacerbation of heart failure.

We observed that more than half of the children were aged 1–5 years, which aligns with previous studies conducted in India (3) and Ethiopia (13), with a comparable median age at diagnosis. On the contrary, a study conducted in North America (5) reported that more than half of their participants were under one year old. This discrepancy could be attributed to differences in pathways to care, advancements in diagnostic tools, and treatment options at their ends.

In our study, half of the affected children had LVEF of 30% and below at the time of diagnosis, with a quarter returning to normal function during the follow-up visits. This finding is similar to a cardiomyopathy registry from the USA and Canada, which reports 22% of the enrolled children recovered to normal cardiac function within 2 years of diagnosis (11, 12). Additionally, in both studies, the commonest cause of DCM was documented to be myocarditis (clinically diagnosed), although in nearly half of the affected children in our cohort, the etiologies were not known. One of the reasons is the limited diagnostic tools in our settings, particularly genetic screening and routine cardiac MRI. This observation was also highlighted in other studies conducted in Nigeria and Ethiopia (13, 16).

During hospital stay, most of the enrolled children received combination therapy, including ACE inhibitors, digoxin, and diuretics, some with beta blockers, while others received inotropic support due to their critical states. In addition, among those with arrhythmias, only one had successful electrical cardioversion with recovery of LVEF to normal upon follow-up visits. Upon initiation of the treatment, most of the children recovered from acute decompensated heart failure. In review of the received treatment of this cohort, we observed a lack of advanced heart failure therapy, including ventricular assist devices and heart transplantation. Also, limited availability of approved heart failure medications, such as Angiotensin Receptor-Neprilysin Inhibitor (ARNI) (17). However, currently, there is no local guideline for the treatment of heart failure in children with DCM, which underscores the need to develop one for the betterment of the quality of life of these affected children.

At the time of discharge, three children died out of 35 children. During the out-of-hospital follow-up, an additional six children died. Those who died in the hospital setting were in critically ill states that needed admission to the intensive care unit (ICU), while for those who died post discharge in the peripheral hospital, the exact causes of their deaths were not well established. However, of these six children, some were reported to have had symptoms of acute gastroenteritis before their demise. Despite the small number of our cohort, the observed number of children who died is significant, which is comparable to a study done in Nigeria (16). Studies in India and Ethiopia documented a lower proportion (3, 13). This disparity in proportions may be partly explained by the differences in the number of study participants and in the etiological profile. For instance, in the study conducted in Ethiopia, most of their participants had an unknown cause for DCM, while the one in India had a sample size four times higher than ours.

Despite the significant findings, this study had some limitations, including its retrospective design, been prone to incomplete or missing information such as such as LV dimensions, z-scores. Secondly, the small number of study participants and loss to follow-up limited further meaningful statistical analysis. Thirdly, this was a single-center study conducted at a national tertiary cardiac referral center; the findings may not be generalizable to other healthcare settings. Lastly, limited access to advanced diagnostic investigations, including cardiac MRI, genetic testing and endomyocardial biopsy.

## Conclusion

Children aged below 5 years were largely affected in our cohort, with clinically diagnosed myocarditis as the most common underlying cause. A quarter of the children showed complete recovery of heart function during the follow-up visits. We observed mortality and in-hospital complications such as stroke and AKI. Further studies involving genetic screening to elucidate the possible causes for the children with an unknown etiology of DCM are highly recommended.

## Data Availability

The original contributions presented in the study are included in the article/Supplementary Material, further inquiries can be directed to the corresponding author.
